# The top 100 most‐cited knee osteotomy publications

**DOI:** 10.1002/jeo2.70175

**Published:** 2025-04-01

**Authors:** Matthieu Ollivier, Jean‐François Gonzalez, Axel Machado, Sachin Tapasvi, Raghbir Khakha, Ronald van Heerwaarden, Grégoire Micicoi

**Affiliations:** ^1^ Department of Orthopedics and Traumatology Institute of Movement and Locomotion, St. Marguerite Hospital Marseille France; ^2^ IULS‐University Institute for Locomotion and Sports, ICARE Laboratory, Pasteur 2 Hospital Nice France; ^3^ Orthopaedic Specialty Clinic Pune India; ^4^ Guys and St Thomas' Hospitals London UK; ^5^ Centre for Deformity Correction and Joint Preserving Surgery, Kliniek ViaSana Mill the Netherlands

**Keywords:** 100 most‐cited articles, bibliometric, distal femoral osteotomy, high tibial osteotomy, knee osteotomy

## Abstract

**Purpose:**

To objectively identify the 100 most influential scientific publications in knee osteotomy and provide an analysis of their main characteristics.

**Methods:**

The Clarivate Analytics Web of Knowledge database was used to obtain data and metrics on knee osteotomy research. The search list was sorted by the number of citations, and articles were included or excluded based on relevance to knee osteotomy. The information extracted for each article included the author's name, publication year, country of origin, journal name, article type and the level of evidence.

**Results:**

These 100 studies generated a total of 16,246 citations, with an average of 162.5 citations per article. The most‐cited article was cited 752 times. The 100 studies included in this analysis were published between 1976 and 2015. Twenty‐one different journals published these 100 publications. The majority of the publications were from the United States (*n* = 30), followed by Germany (*n* = 17) and Japan (*n* = 11). The most prevalent study designs were case series (*n* = 55) and cohort studies (*n* = 19).

**Conclusion:**

The 100 most influential publications in knee osteotomy were cited a total of 16,246 times. The study designs most used were case series and cohort studies with low‐level evidence. This publication serves as a reference to direct orthopaedic practitioners to the 100 most influential studies in knee osteotomy and target future research directions.

**Clinical Relevance:**

This analysis of the 100 most influential (or cited) scientific publications in osteotomy around the knee will provide a comprehensive inventory of the most impactful academic contributions to a field that has recently regained interest among medical students, residents, fellows and attending physicians.

**Level of Evidence:**

N/A.

**Abbreviations**
ISAKOS: International Society of Arthroscopy, Knee Surgery and Orthopaedic Sports Medicine
PSI: patient‐specific instrumentation

## INTRODUCTION

Osteotomy around the knee was once a well‐established technique for the treatment of unicompartmental osteoarthritis of the knee [11]. This procedure was later slowly replaced by total and unicompartmental knee arthroplasty as it was considered a more demanding surgical procedure with unpredictable outcomes [7]. Nevertheless, the indications for knee osteotomy and arthroplasty are different considering the deformity, osteoarthritis stage and patient activity. The first guidelines for indications for osteotomy were proposed by The International Society of Arthroscopy, Knee Surgery and Orthopaedic Sports Medicine (ISAKOS) in 2005 [28].

There is a recent renewed scientific interest in knee osteotomy pertaining to newer materials as well as accuracy tools like patient‐specific instrumentation (PSI) [9, 13, 22]. We now have a better understanding of the correction target (risk of overcorrecting the tibia or leaving a residual femoral deformity) [2, 16, 25]. This has led to an approach which tends to perform more double‐level osteotomy in such deformities to retain the joint line obliquity, improve functional outcomes [6, 14, 17, 29] and improve the long‐term survival rates [3, 5].

The resulting increased number of publications on osteotomy around the knee makes it difficult to develop a comprehensive foundation in the literature, as this requires orthopaedic residents, fellows, and surgeons to prioritize the most important studies. In this aspect, citation analysis has been shown to be an effective tool in identifying impactful articles [10, 15]. Citation analyses objectively identify publications that help offer valuable insight into the history and evolution of a specialized technique. The most‐cited literature on knee osteotomy is expected to reflect the trends and growth of the technique itself. Surgeons who practice knee osteotomy can use these publications to examine the characteristics of the most‐cited studies in osteotomy around the knee, have a better understanding of the approach over time, and focus on their research contributions. Currently, citation analyses are available on topics including knee research [1], hip and knee arthroplasty [18, 20, 21, 32], knee arthroscopy [26] and anterior cruciate ligament reconstruction [33]. There is a lack of literature on the results of double‐level osteotomies on functional outcomes and long‐term survival. Understanding the path taken by subsequent studies to lead to this reflection on the benefits of double‐level osteotomies and deformity analysis is essential.

The purpose of this article was to objectively identify the 100 most influential publications in osteotomy around the knee and provide an analysis of their key characteristics. This study hypothesized that many of the most‐cited studies were published in the last 20 years.

## METHODS

An institutional review board was not necessary for this study as all data is available in the public domain. Data was procured from the Clarivate Analytics Web of Knowledge database using Boolean queries [18, 26, 30]. While no citation tracking service is perfect, the Clarivate Analytics Web of Knowledge represents a very extensive database that covers more than 21,000 peer‐reviewed scholarly journals. Its database is also noted for its high‐quality citation links, accuracy, comprehensive coverage, and consistent use among numerous previous citation analyses [31].

The initial database search took place in April 2024 incorporating various Boolean search terms to capture all possible iterations of osteotomy around the knee. The search strategy included ‘Knee osteotomy’ OR ‘High Tibial Osteotomy’ OR ‘Distal Femoral Osteotomy’ OR ‘Double Level Osteotomy’. The search was carried out with no limitations on date of publication, journal, or country of origin. However, only publications in English were included. This resulted in a total of 9766 articles.

The list of publications was organized by the total number of citations in descending order. The title and abstract of each publication were reviewed to determine its relevance to osteotomy around the knee. To qualify for selection, the publication had to present information on surgical outcomes, surgical indications, descriptions of procedures or complications after osteotomy around the knee. If the publication did not meet any of these inclusion criteria, it was excluded. In addition, knee osteotomy had to be the focus of the study to be included. If the publication made peripheral mention of knee osteotomy, it was excluded. For example, any study that analyzed the different treatment options for osteoarthritis, including medical treatment, was excluded. Articles concerning tibial tuberosity osteotomy were excluded as the analysis concerned osteotomy for malalignment correction. If the inclusion of a study was in question after review of the title and abstract, a copy of the full article was obtained and reviewed by two authors (GM and RK) to decide upon inclusion or exclusion.

A total of 327 of the most‐cited articles were reviewed to reach the 100 most‐cited studies that met the designated inclusion criteria. The author's country of origin, number of citations, journal title, year of publication, and study design (laboratory study, review article, descriptive study, case report, case series, cohort study, case‐control and randomized controlled trial) were extracted. The level of evidence with the article's relative risk of bias was determined based on guidelines published in The Arthroscopy Journal [19]. The study design and level of evidence were classified by the consensus opinion between two authors (GM and RK). If a consensus could not be obtained, the senior author (MO) was consulted for the final decision. The final list of the 100 most‐cited articles was then organized based on total citations and presented in descending order. Citation density was then calculated by the total number of citations divided by the years since the paper was published [20]. If two articles had the same position based on total citation, the higher citation density was placed first.

## RESULTS

**Figure 1:**
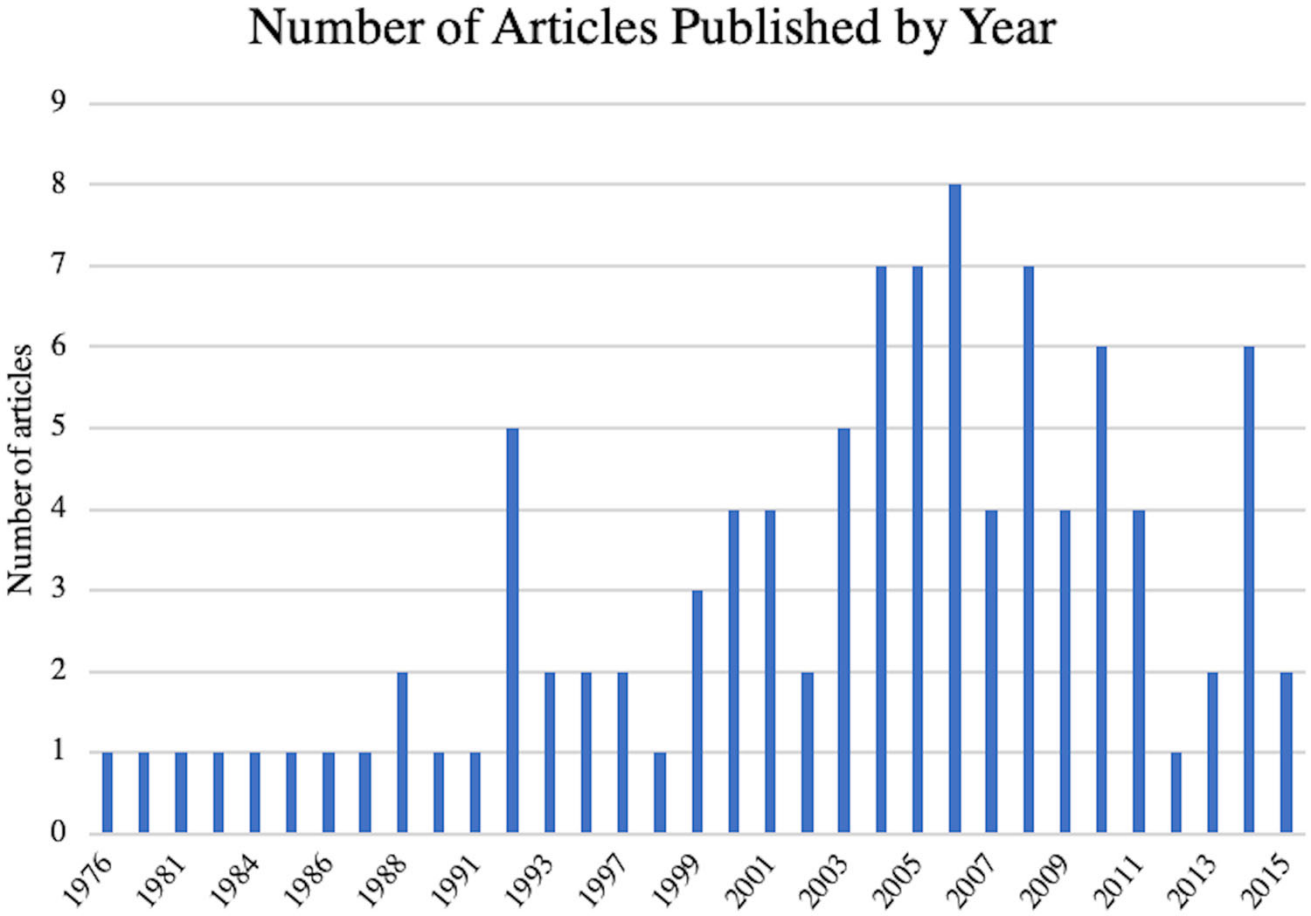
The number of most‐cited 100 osteotomy around the knee publications per year.

The 100 most‐cited osteotomy around the knee articles were published between 1976 and 2015. Most of the publications were after 1992, and approximately one half were published in the 2000s. Four years, 2004, 2005, 2006 and 2008, were particularly productive, with seven or more publications every year (Figure 1). In total, the number of citations for these 100 publications numbered 16,246. This averaged 162.5 citations per paper. The total number of citations for the top 100 articles ranged from 84 to 752 (Table 1). Citation density was analyzed in addition to total citation. The most‐cited publication in 2002 amassed 752 citations. It was also the most citation dense, averaging 37.6 citations per year. The least citation‐dense article with an average of 2.2 citations per year, was the oldest article in our study (1976) with a total of 103 total citations at the time of this analysis.

**Table 1:** The Top 100 cited osteotomy around the knee publications.

Rank	Article	No. of citations (citation density*)	Original publication year	Study design
1	Wakitani S, Imoto K, Yamamoto T, Saito M, Murata N, Yoneda M. Human Autologous Culture Expanded Bone Marrow Mesenchymal Cell Transplantation For Repair Of Cartilage Defects In Osteoarthritic Knees. Osteoarthritis Cartilage 2002	752 (37.6)	2002	Randomized controlled trial
2	Fujisawa Y, Masuhara K, Shiomi S. Effect Of High Tibial Osteotomy On Osteoarthritis Of The Knee ‐ Arthroscopic Study Of 54 Knee Joints. Orthop. Clin. North Am. 1979	510 (11.9)	1979	Cohort study
3	Coventry Mb, Ilstrup Dm, Wallrichs Sl. Proximal Tibial Osteotomy ‐ A Critical Long‐Term Study Of 87 Cases. J. Bone Joint Surg.‐Am. Vol. 1993	475 (16.4)	1993	Case series
4	Giffin Jr, Vogrin Tm, Zantop T, Woo Sly, Harner Cd. Effects Of Increasing Tibial Slope On The Biomechanics Of The Knee. Am. J. Sports Med. 2004	448 (24.9)	2004	Laboratory study
5	Insall Jn, Joseph Dm, Msika C. High Tibial Osteotomy For Varus Gonarthrosis ‐ A Long‐Term Follow‐Up‐Study. J. Bone Joint Surg.‐Am. Vol. 1984	436 (11.5)	1984	Case series
6	Lobenhoffer P, Agneskirchner Jd. Improvements In Surgical Technique Of Valgus High Tibial Osteotomy. Knee Surg. Sports Traumatol. Arthrosc. 2003	360 (18.9)	2003	Case series
7	Prodromos Cc, Andriacchi Tp, Galante Jo. A Relationship Between Gait And Clinical Changes Following High Tibial Osteotomy. J. Bone Joint Surg.‐Am. Vol. 1985	342 (9.2)	1985	Cohort study
8	Dugdale Tw, Noyes Fr, Styer D. Preoperative Planning For High Tibial Osteotomy ‐ The Effect Of Lateral Tibiofemoral Separation And Tibiofemoral Length. Clin. Orthop. Rel. Res. 1992	313 (10.4)	1992	Descriptive article
9	Staubli Ae, De Simoni C, Babst R, Lobenhoffer P. Tomofix: A New Lcp‐Concept For Open Wedge Osteotomy Of The Medial Proximal Tibia ‐ Early Results In 92 Cases. Injury‐Int. J. Care Inj. 2003	283 (14.9)	2003	Case series
10	Geesink Rgt, Hoefnagels Nhm, Bulstra Sk. Osteogenic Activity Of Op‐1 Bone Morphogenetic Protein (Bmp‐7) In A Human Fibular Defect. J. Bone Joint Surg.‐Br. Vol. 1999	278 (12.1)	1999	Randomized controlled trial
11	Verdonk Peter C. M., Verstraete Koenraad L., Almqvist Karl F., De Cuyper Kristof, Veys Eric M., Verbruggen Gust, Verdonk Rene. Meniscal Allograft Transplantation: Long‐Term Clinical Results With Radiological And Magnetic Resonance Imaging Correlations. Knee Surg. Sports Traumatol. Arthrosc. 2006	252 (15.8)	2006	Case series
12	Sprenger Tr, Doerzbacher Je. Tibial Osteotomy For The Treatment Of Varus Gonarthrosis ‐ Survival And Failure Analysis To Twenty‐Two Years. J. Bone Joint Surg.‐Am. Vol. 2003	249 (13.1)	2003	Cohort study
13	Naudie D, Bourne Rb, Rorabeck Ch, Bourne Tj. Survivorship Of The High Tibial Valgus Osteotomy ‐ A 10‐To 22‐Year Followup Study. Clin. Orthop. Rel. Res. 1999	247 (10.7)	1999	Cohort study
14	Wong Keng Lin, Lee Kevin Boon Leng, Tai Bee Choo, Law Ping, Lee Eng Hin, Hui James H. P. Injectable Cultured Bone Marrow‐Derived Mesenchymal Stem Cells In Varus Knees With Cartilage Defects Undergoing High Tibial Osteotomy: A Prospective Randomized Controlled Clinical Trial With 2 Years' Follow‐Up. Arthroscopy 2013	237 (26.3)	2013	Randomized controlled trial
15	Marti Cb, Gautier E, Wachtl Sw, Jakob Rp. Accuracy Of Frontal And Sagittal Plane Correction In Open‐Wedge High Tibial Osteotomy. Arthroscopy 2004	218 (12.1)	2004	Case series
16	Brinkman J. ‐M., Lobenhoffer P., Agneskirchner J. D., Staubli A. E., Wymenga A. B., Van Heerwaarden R. J. Osteotomies Around The Knee Patient Selection Stability Of Fixation And Bone Healing In High Tibial Osteotomies. J. Bone Joint Surg.‐Br. Vol. 2008	213 (15.2)	2008	Descriptive article
17	Noyes Fr, Barber‐Westin Sd, Hewett Te. High Tibial Osteotomy And Ligament Reconstruction For Varus Angulated Anterior Cruciate Ligament‐Deficient Knees. Am. J. Sports Med. 2000	213 (9.7)	2000	Case series
18	Amendola Annunziato, Bonasia Davide Edoardo. Results Of High Tibial Osteotomy: Review Of The Literature. Int. Orthop. 2010	209 (17.4)	2010	Review article
19	Akizuki S., Shibakawa A., Takizawa T., Yamazaki I., Horiuchi H. The Long‐Term Outcome Of High Tibial Osteotomy ‐ A Ten‐ To 20‐Year Follow‐Up. J. Bone Joint Surg.‐Br. Vol. 2008	202 (14.4)	2008	Case series
20	Agneskirchner Jens Dominik, Hurschler Christof, Wrann Christiane D., Lobenhoffer Philipp. The Effects Of Valgus Medial Opening Wedge High Tibial Osteotomy On Articular Cartilage Pressure Of The Knee: A Biomechanical Study. Arthroscopy 2007	202 (13.5)	2007	Laboratory study
21	Agneskirchner Jd, Hurschler C, Stukenborg‐Colsman C, Imhoff Ab, Lobenhoffer P. Effect Of High Tibial Flexion Osteotomy On Cartilage Pressure And Joint Kinematics: A Biomechanical Study In Human Cadaveric Knees ‐ Winner Of The Aga‐Donjoy Award 2004. Arch. Orthop. Trauma Surg. 2004	200 (11.1)	2004	Laboratory study
22	Paley D, Tetsworth K. Mechanical Axis Deviation Of The Lower‐Limbs ‐ Preoperative Planning Of Uniapical Angular Deformities Of The Tibia Or Femur. Clin. Orthop. Rel. Res. 1992	198 (6.6)	1992	Descriptive article
23	Leclerc Jr, Geerts Wh, Desjardins L, Jobin F, Laroche F, Delorme F, Haviernick S, Atkinson S, Bourgouin J. Prevention Of Deep‐Vein Thrombosis After Major Knee Surgery ‐ A Randomized Double‐Blind Trial Comparing A Low‐Molecular‐Weight Heparin Fragment (Enoxaparin) To Placebo. Thromb. Haemost. 1992	194 (6.5)	1992	Case series
24	Koshino T, Murase T, Saito T. Medial Opening‐Wedge High Tibial Osteotomy With Use Of Porous Hydroxyapatite To Treat Medial Compartment Osteoarthritis Of The Knee. J. Bone Joint Surg.‐Am. Vol. 2003	187 (9.8)	2003	Case series
25	Minas T. Autologous Chondrocyte Implantation For Focal Chondral Defects Of The Knee. Clin. Orthop. Rel. Res. 2001	184 (8.8)	2001	Cohort study
26	Beaver Rj, Mahomed M, Backstein D, Davis A, Zukor Dj, Gross Ae. Fresh Osteochondral Allografts For Posttraumatic Defects In The Knee ‐ A Survivorship Analysis. J. Bone Joint Surg.‐Br. Vol. 1992	181 (6)	1992	Case series
27	Floerkemeier Stephanie, Staubli Alex E., Schroeter Steffen, Goldhahn Sabine, Lobenhoffer Philipp. Outcome After High Tibial Open‐Wedge Osteotomy: A Retrospective Evaluation Of 533 Patients. Knee Surg. Sports Traumatol. Arthrosc. 2013	179 (19.9)	2013	Cohort study
28	Billings A, Scott Df, Camargo Mp, Hofmann Aa. High Tibial Osteotomy With A Calibrated Osteotomy Guide Rigid Internal Fixation And Early Motion ‐ Long‐Term Follow‐Up. J. Bone Joint Surg.‐Am. Vol. 2000	179 (8.1)	2000	Case series
29	Noyes Fr, Goebel Sx, West J. Opening Wedge Tibial Osteotomy ‐ The 3‐Triangle Method To Correct Axial Alignment And Tibial Slope. Am. J. Sports Med. 2005	172 (10.1)	2005	Laboratory study
30	Noyes Fr, Barber‐Westin Sd. Revision Anterior Cruciate Surgery With Use Of Bone‐Patellar Tendon‐Bone Autogenous Grafts. J. Bone Joint Surg.‐Am. Vol. 2001	167 (8)	2001	Case series
31	Noyes Fr, Barber Sd, Simon R. High Tibial Osteotomy And Ligament Reconstruction In Varus Angulated Anterior Cruciate Ligament‐Deficient Knees ‐ A 2‐Year To 7‐Year Follow‐Up‐Study. Am. J. Sports Med. 1993	156 (5.4)	1993	Case series
32	Miller Bruce S., Downie Brian, Mcdonough E. Barry, Wojtys Edward M. Complications After Medial Opening Wedge High Tibial Osteotomy. Arthroscopy 2009	155 (11.9)	2009	Case series
33	Agneskirchner Jd, Freiling D, Hurschler C, Lobenhoffer P. Primary Stability Of Four Different Implants For Opening Wedge High Tibial Osteotomy. Knee Surg. Sports Traumatol. Arthrosc. 2006	154 (9.6)	2006	Laboratory study
34	Brouwer R. W., Bierma‐Zeinstra S. M. A., Van Raaij T. M., Verhaar J. A. N. Osteotomy For Medial Compartment Arthritis Of The Knee Using A Closing Wedge Or An Opening Wedge Controlled By A Puddu Plate ‐ A One‐Year Randomised Controlled Study. J. Bone Joint Surg.‐Br. Vol. 2006	153 (9.6)	2006	Randomized controlled trial
35	Stoffel K, Stachowiak G, Kuster M. Open Wedge High Tibial Osteotomy: Biomechanical Investigation Of The Modified Arthrex Steotomy Plate (Puddu Plate) And The Tomofix Plate. Clin. Biomech. 2004	151 (8.4)	2004	Laboratory study
36	Ivarsson I, Myrnerts R, Gillquist J. High Tibial Osteotomy For Medial Osteoarthritis Of The Knee ‐ A 5 To 7 And An 11 To 13 Year Follow‐Up. J. Bone Joint Surg.‐Br. Vol. 1990	148 (4.6)	1990	Case series
37	Takeuchi Ryohei, Ishikawa Hiroyuki, Kumagai Ken, Yamaguchi Yuichiro, Chiba Naoki, Akamatsu Yasushi, Saito Tomoyuki. Fractures Around The Lateral Cortical Hinge After A Medial Opening‐Wedge High Tibial Osteotomy: A New Classification Of Lateral Hinge Fracture. Arthroscopy 2012	146 (14.6)	2012	Case series
38	Cameron Jc, Saha S. Meniscal Allograft Transplantation For Unicompartmental Arthritis Of The Knee. Clin. Orthop. Rel. Res. 1997	146 (5.8)	1997	Case series
39	Dallari D., Savarino L., Stagni C., Cenni E., Cenacchi A., Fornasari P. M., Albisinni U., Rimondi E., Baldini N., Giunti A. Enhanced Tibial Osteotomy Healing With Use Of Bone Grafts Supplemented With Platelet Gel Or Platelet Gel And Bone Marrow Stromal Cells. J. Bone Joint Surg.‐Am. Vol. 2007	145 (9.7)	2007	Randomized controlled trial
40	Brouwer Rw, Bierma‐Zeinstra Sma, Van Koeveringe Aj, Verhaar Jan. Patellar Height And The Inclination Of The Tibial Plateau After High Tibial Osteotomy ‐ The Open Versus The Closed‐Wedge Technique. J. Bone Joint Surg.‐Br. Vol. 2005	144 (8.5)	2005	Randomized controlled trial
41	Minas Tom, Von Keudell Arvind, Bryant Tim, Gomoll Andreas H. The John Insall Award: A Minimum 10‐Year Outcome Study Of Autologous Chondrocyte Implantation. Clin. Orthop. Rel. Res. 2014	143 (17.9)	2014	Case series
42	Koshino T. The Treatment Of Spontaneous Osteonecrosis Of The Knee By High Tibial Osteotomy With And Without Bone‐Grafting Or Drilling Of The Lesion. J. Bone Joint Surg.‐Am. Vol. 1982	140 (3.5)	1982	Case series
43	Takeuchi Ryohei, Ishikawa Hiroyuki, Aratake Masato, Bito Haruhiko, Saito Izumi, Kumagai Ken, Akamatsu Yasuhsi, Saito Tomoyuki. Medial Opening Wedge High Tibial Osteotomy With Early Full Weight Bearing. Arthroscopy 2009	138 (10.6)	2009	Case series
44	Paley D. Principles Of Deformity Correction Around The Knee. Orthopade 2000	133 (6)	2000	Descriptive article
45	Vainionpaa S, Laike E, Kirves P, Tiusanen P. Tibial Osteotomy For Osteo‐Arthritis Of The Knee ‐ A 5 To 10‐Year Follow‐Up‐Study. J. Bone Joint Surg.‐Am. Vol. 1981	133 (3.2)	1981	Case series
46	Hui Catherine, Salmon Lucy J., Kok Alison, Williams Heidi A., Hockers Niels, Van Der Tempel Willem M., Chana Rishi, Pinczewski Leo A. Long‐Term Survival Of High Tibial Osteotomy For Medial Compartment Osteoarthritis Of The Knee. Am. J. Sports Med. 2011	131 (11.9)	2011	Case series
47	Koshino T, Wada S, Ara Y, Saito T. Regeneration Of Degenerated Articular Cartilage After High Tibial Valgus Osteotomy For Medial Compartmental Osteoarthritis Of The Knee. Knee 2003	131 (6.9)	2003	Case series
48	Koshino T, Yoshida T, Ara Y, Saito I, Saito T. Fifteen To Twenty‐Eight Years' Follow‐Up Results Of High Tibial Valgus Osteotomy For Osteoarthritic Knee. Knee 2004	130 (7.2)	2004	Case series
49	Gaasbeek Rda, Toonen Hg, Van Heerwaarden Rj, Buma P. Mechanism Of Bone Incorporation Of Beta‐Tcp Bone Substitute In Open Wedge Tibial Osteotomy In Patients. Biomaterials 2005	129 (7.6)	2005	Case series
50	Coventry Mb. Proximal Tibial Varus Osteotomy For Osteoarthritis Of The Lateral Compartment Of The Knee. J. Bone Joint Surg.‐Am. Vol. 1987	129 (3.7)	1987	Case series
51	Koh Yong‐Gon, Kwon Oh‐Ryong, Kim Yong‐Sang, Choi Yun‐Jin. Comparative Outcomes Of Open‐Wedge High Tibial Osteotomy With Platelet‐Rich Plasma Alone Or In Combination With Mesenchymal Stem Cell Treatment: A Prospective Study. Arthroscopy 2014	128 (16)	2014	Cohort study
52	Rodner Craig M., Adams Douglas J., Diaz‐Doran Vilmaris, Tate Janet P., Santangelo Stephen A., Mazzocca Augustus D., Arciero Robert A. Medial Opening Wedge Tibial Osteotomy And The Sagittal Plane ‐ The Effect Of Increasing Tibial Slope On Tibiofemoral Contact Pressure. Am. J. Sports Med. 2006	127 (7.9)	2006	Laboratory study
53	Yasuda K, Majima T, Tsuchida T, Kaneda K. A 10‐Year To 15‐Year Follow‐Up Observation Of High Tibial Osteotomy In Medial Compartment Osteoarthrosis. Clin. Orthop. Rel. Res. 1992	125 (4.2)	1992	Case series
54	Berman At, Bosacco Sj, Kirshner S, Avolio A. Factors Influencing Long‐Term Results In High Tibial Osteotomy. Clin. Orthop. Rel. Res. 1991	125 (4)	1991	Review article
55	Flecher Xavier, Parratte Sebastien, Aubaniac Jean‐Manuel, Argenson Jean‐Noel A. A 12‐28‐Year Followup Study Of Closing Wedge High Tibial Osteotomy. Clin. Orthop. Rel. Res. 2006	123 (7.7)	2006	Case series
56	Broughton Ns, Newman Jh, Baily Raj. Unicompartmental Replacement And High Tibial Osteotomy For Osteoarthritis Of The Knee ‐ A Comparative‐Study After 5‐10 Years Follow‐Up. J. Bone Joint Surg.‐Br. Vol. 1986	123 (3.4)	1986	Cohort study
57	Niemeyer Philipp, Schmal Hagen, Hauschild Oliver, Von Heyden Johanna, Suedkamp Norbert P., Koestler Wolfgang. Open‐Wedge Osteotomy Using An Internal Plate Fixator In Patients With Medial‐Compartment Gonarthritis And Varus Malalignment: 3‐Year Results With Regard To Preoperative Arthroscopic And Radiographic Findings. Arthroscopy 2010	122 (10.2)	2010	Case series
58	Giffin J. Robert, Stabile Kathryne J., Zantop Thore, Vogrin Tracy M., Woo Savio L‐Y., Harner Christopher D. Importance Of Tibial Slope For Stability Of The Posterior Cruciate Ligament‐Deficient Knee. Am. J. Sports Med. 2007	120 (8)	2007	Laboratory study
59	Dejour David, Saffarini Mo, Demey Guillaume, Baverel Laurent. Tibial Slope Correction Combined With Second Revision Acl Produces Good Knee Stability And Prevents Graft Rupture. Knee Surg. Sports Traumatol. Arthrosc. 2015	117 (1.7)	2015	Cohort study
60	Stukenborg‐Colsman C, Wirth Cj, Lazovic D, Wefer A. High Tibial Osteotomy Versus Unicompartmental Joint Replacement In Unicompartmental Knee Joint Osteoarthritis: 7‐10‐Year Follow‐Up Prospective Randomised Study. Knee 2001	115 (5.5)	2001	Cohort study
61	W‐Dahl Annette, Robertsson Otto, Lidgren Lars. Surgery For Knee Osteoarthritis In Younger Patients. Acta Orthop. 2010	112 (9.3)	2010	Cohort study
62	Hankemeier S., Hufner T., Wang G., Kendoff D., Zeichen J., Zheng G., Krettek C. Navigated Open‐Wedge High Tibial Osteotomy: Advantages And Disadvantages Compared To The Conventional Technique In A Cadaver Study. Knee Surg. Sports Traumatol. Arthrosc. 2006	111 (6.9)	2006	Laboratory study
63	Rosenberger Ralf E., Gomoll Andreas H., Bryant Tim, Minas Tom. Repair Of Large Chondral Defects Of The Knee With Autologous Chondrocyte Implantation In Patients 45 Years Or Older. Am. J. Sports Med. 2008	110 (7.9)	2008	Case series
64	Jung Woon‐Hwa, Takeuchi Ryohei, Chun Chung‐Woo, Lee Jung‐Su, Ha Jae‐Hun, Kim Ji‐Hyae, Jeong Jae‐Heon. Second‐Look Arthroscopic Assessment Of Cartilage Regeneration After Medial Opening‐Wedge High Tibial Osteotomy. Arthroscopy 2014	109 (13.6)	2014	Case series
65	El‐Azab Hosam, Glabgly Parpakorn, Paul Jochen, Imhoff Andreas B., Hinterwimmer Stefan. Patellar Height And Posterior Tibial Slope After Open‐ And Closed‐Wedge High Tibial Osteotomy A Radiological Study On 100 Patients. Am. J. Sports Med. 2010	107 (8.9)	2010	Cohort study
66	Wright Jm, Crockett Hc, Slawski Dp, Madsen Mw, Windsor Re. High Tibial Osteotomy. J. Am. Acad. Orthop. Surg. 2005	107 (6.3)	2005	Review article
67	Akizuki S, Yasukawa Y, Takizawa T. Does Arthroscopic Abrasion Arthroplasty Promote Cartilage Regeneration In Osteoarthritic Knees With Eburnation? A Prospective Study Of High Tibial Osteotomy With Abrasion Arthroplasty Versus High Tibial Osteotomy Alone. Arthroscopy 1997	107 (4.3)	1997	Cohort study
68	Gaasbeek Robert D. A., Nicolaas Loes, Rijnberg Willard J., Van Loon Corne J. M., Van Kampen Albert. Correction Accuracy And Collateral Laxity In Open Versus Closed Wedge High Tibial Osteotomy. A One‐Year Randomised Controlled Study. Int. Orthop. 2010	105 (8.8)	2010	Randomized controlled trial
69	Nelissen E. M., Van Langelaan E. J., Nelissen R. G. H. H. Stability Of Medial Opening Wedge High Tibial Osteotomy: A Failure Analysis. Int. Orthop. 2010	105 (8.8)	2010	Case series
70	Weale Ae, Newman Jh. Unicompartmental Arthroplasty And High Tibial Osteotomy For Osteoarthrosis Of The Knee ‐ A Comparative‐Study With A 12‐Year To 17‐Year Follow‐Up Period. Clin. Orthop. Rel. Res. 1994	105 (3.8)	1994	Cohort study
71	Bonasia Davide Edoardo, Dettoni Federico, Sito Gabriele, Blonna Davide, Marmotti Antongiulio, Bruzzone Matteo, Castoldi Filippo, Rossi Roberto. Medial Opening Wedge High Tibial Osteotomy For Medial Compartment Overload/Arthritis In The Varus Knee Prognostic Factors. Am. J. Sports Med. 2014	104 (13)	2014	Case series
72	Sonnery‐Cottet Bertrand, Mogos Stefan, Thaunat Mathieu, Archbold Pooler, Fayard Jean‐Marie, Freychet Benjamin, Clechet Julien, Chambat Pierre. Proximal Tibial Anterior Closing Wedge Osteotomy In Repeat Revision Of Anterior Cruciate Ligament Reconstruction. Am. J. Sports Med. 2014	104 (13)	2014	Case series
73	Dejour H, Neyret P, Boileau P, Donell St. Anterior Cruciate Reconstruction Combined With Valgus Tibial Osteotomy. Clin. Orthop. Rel. Res. 1994	103 (3.7)	1994	Case series
74	Maquet P. Valgus Osteotomy For Osteoarthritis Of Knee. Clin. Orthop. Rel. Res. 1976	103 (2.2)	1976	Case series
75	Meidinger Gebhart, Imhoff Andreas B., Paul Jochen, Kirchhoff Chlodwig, Sauerschnig Martin, Hinterwimmer Stefan. May Smokers And Overweight Patients Be Treated With A Medial Open‐Wedge Hto? Risk Factors For Non‐Union. Knee Surg. Sports Traumatol. Arthrosc. 2011	102 (9.3)	2011	Case series
76	Birmingham Trevor B., Giffin J. Robert, Chesworth Bert M., Bryant Dianne M., Litchfield Robert B., Willits Kevin, Jenkyn Thomas R., Fowler Peter J. Medial Opening Wedge High Tibial Osteotomy: A Prospective Cohort Study Of Gait Radiographic And Patient‐Reported Outcomes. Arthritis Rheum‐Arthritis Care Res. 2009	99 (7.6)	2009	Cohort study
77	Healy Wl, Anglen Jo, Wasilewski Sa, Krackow Ka. Distal Femoral Varus Osteotomy. J. Bone Joint Surg.‐Am. Vol. 1988	99 (2.9)	1988	Case series
78	Schallberger Alex, Jacobi Matthias, Wahl Peter, Maestretti Gianluca, Jakob Roland P. High Tibial Valgus Osteotomy In Unicompartmental Medial Osteoarthritis Of The Knee: A Retrospective Follow‐Up Study Over 13‐21 Years. Knee Surg. Sports Traumatol. Arthrosc. 2011	98 (8.9)	2011	Case series
79	Lobenhoffer P, Agneskirchner J, Zoch W. Open‐Wedge High Tibial Osteotomy With Special Medial Plate Fixator. Orthopade 2004	98 (5.4)	2004	Case series
80	Saragaglia D, Roberts J. Navigated Osteotomies Around The Knee In 170 Patients With Osteoarthritis Secondary To Genu Varum. Orthopedics 2005	97 (5.7)	2005	Cohort study
81	Niemeyer Philipp, Koestler Wolfgang, Kaehny Christian, Kreuz Peter C., Brooks Christopher J., Strohm Peter C., Helwig Peter, Suedkamp Norbert P. Two‐Year Results Of Open‐Wedge High Tibial Osteotomy With Fixation By Medial Plate Fixator For Medial Compartment Arthritis With Varus Malalignment Of The Knee. Arthroscopy 2008	96 (6.9)	2008	Case series
82	Martin Robin, Birmingham Trevor B., Willits Kevin, Litchfield Robert, Lebel Marie‐Eve, Giffin J. Robert. Adverse Event Rates And Classifications In Medial Opening Wedge High Tibial Osteotomy. Am. J. Sports Med. 2014	95 (11.9)	2014	Case series
83	El‐Azab H., Halawa A., Anetzberger H., Imhoff A. B., Hinterwimmer S. The Effect Of Closed‐ And Open‐Wedge High Tibial Osteotomy On Tibial Slope ‐ A Retrospective Radiological Review Of 120 Cases. J. Bone Joint Surg.‐Br. Vol. 2008	95 (6.8)	2008	Case series
84	Lonner Jh, Siliski Jm, Lotke Pa. Simultaneous Femoral Osteotomy And Total Knee Arthroplasty For Treatment Of Osteoarthritis Associated With Severe Extra‐Articular Deformity. J. Bone Joint Surg.‐Am. Vol. 2000	95 (4.3)	2000	Case series
85	Salzmann Gian M., Ahrens Philipp, Naal Florian D., El‐Azab Hosam, Spang Jeffrey T., Imhoff Andreas B., Lorenz Stephan. Sporting Activity After High Tibial Osteotomy For The Treatment Of Medial Compartment Knee Osteoarthritis. Am. J. Sports Med. 2009	94 (7.2)	2009	Case series
86	Naudie Ddr, Amendola A, Fowler Pj. Opening Wedge High Tibial Osteotomy For Symptomatic Hyperextension‐Varus Thrust. Am. J. Sports Med. 2004	94 (5.2)	2004	Case series
87	Babis Gc, An Kn, Chao Eys, Rand Ja, Sim Fh. Double Level Osteotomy Of The Knee: A Method To Retain Joint‐Line Obliquity ‐ Clinical Results. J. Bone Joint Surg.‐Am. Vol. 2002	94 (4.7)	2002	Case series
88	Noyes Frank R., Mayfield William, Barber‐Westin Sue D., Albright Jay C., Heckmann Timothy R. Opening Wedge High Tibial Osteotomy ‐ An Operative Technique And Rehabilitation Program To Decrease Complications And Promote Early Union And Function. Am. J. Sports Med. 2006	93 (5.8)	2006	Case series
89	Rinonapoli E, Mancini Gb, Corvaglia A, Musiello S. Tibial Osteotomy For Varus Gonarthrosis ‐ A 10‐ To 21‐Year Followup Study. Clin. Orthop. Rel. Res. 1998	93 (3.9)	1998	Case series
90	Arthur Andrew, Laprade Robert F., Agel Julie. Proximal Tibial Opening Wedge Osteotomy As The Initial Treatment For Chronic Posterolateral Corner Deficiency In The Varus Knee ‐ A Prospective Clinical Study. Am. J. Sports Med. 2007	92 (6.1)	2007	Cohort study
91	Hernigou P, Ma W. Open Wedge Tibial Osteotomy With Acrylic Bone Cement As Bone Substitute. Knee 2001	92 (4.4)	2001	Case series
92	Mcdermott Agp, Finklestein Ja, Farine I, Boynton El, Macintosh Dl, Gross A. Distal Femoral Varus Osteotomy For Valgus Deformity Of The Knee. J. Bone Joint Surg.‐Am. Vol. 1988	91 (2.7)	1988	Case series
93	Bode Gerrit, Von Heyden Johanna, Pestka Jan, Schmal Hagen, Salzmann Gian, Suedkamp Norbert, Niemeyer Philipp. Prospective 5‐Year Survival Rate Data Following Open‐Wedge Valgus High Tibial Osteotomy. Knee Surg. Sports Traumatol. Arthrosc. 2015	90 (12.9)	2015	Case series
94	Hoell S, Suttmoeller J, Stoll V, Fuchs S, Gosheger G. The High Tibial Osteotomy Open Versus Closed Wedge A Comparison Of Methods In 108 Patients. Arch. Orthop. Trauma Surg. 2005	90 (5.3)	2005	Cohort study
95	Magyar G, Ahl Tl, Vibe P, Toksvig‐Larsen S, Lindstrand A. Open‐Wedge Osteotomy By Hemicallotasis Or The Closed‐Wedge Technique For Osteoarthritis Of The Knee ‐ A Randomised Study Of 50 Operations. J. Bone Joint Surg.‐Br. Vol. 1999	90 (3.9)	1999	Randomized controlled trial
96	Brouwer Rw, Jakma Tsc, Bierma‐Zeinstra Sma, Verhagen Ap, Verhaar J. Osteotomy For Treating Knee Osteoarthritis. Cochrane Database Syst Rev. 2005	87 (5.1)	2005	Review article
97	Michaela Gstoettner, Florian Pedross, Michael Liebensteiner, Christian Bach. Long‐Term Outcome After High Tibial Osteotomy. Arch. Orthop. Trauma Surg. 2008	86 (6.1)	2008	Cohort study
98	Spahn G, Kirschbaum S, Kahl E. Factors That Influence High Tibial Osteotomy Results In Patients With Medial Gonarthritis: A Score To Predict The Results. Osteoarthritis Cartilage 2006	86 (5.4)	2006	Case series
99	Brosset T., Pasquier G., Migaud H., Gougeon F. Opening Wedge High Tibial Osteotomy Performed Without Filling The Defect But With Locking Plate Fixation (Tomofix (Tm)) And Early Weight‐Bearing: Prospective Evaluation Of Bone Union Precision And Maintenance Of Correction In 51 Cases. Orthop. Traumatol.‐Surg. Res. 2011	84 (7.6)	2011	Case series
100	Mina Curtis, Garrett William E., Pietrobon Ricardo, Glisson Richard, Higgins Laurence. High Tibial Osteotomy For Unloading Osteochondral Defects In The Medial Compartment Of The Knee. Am. J. Sports Med. 2008	84 (6)	2008	Laboratory study

**Figure 2:**
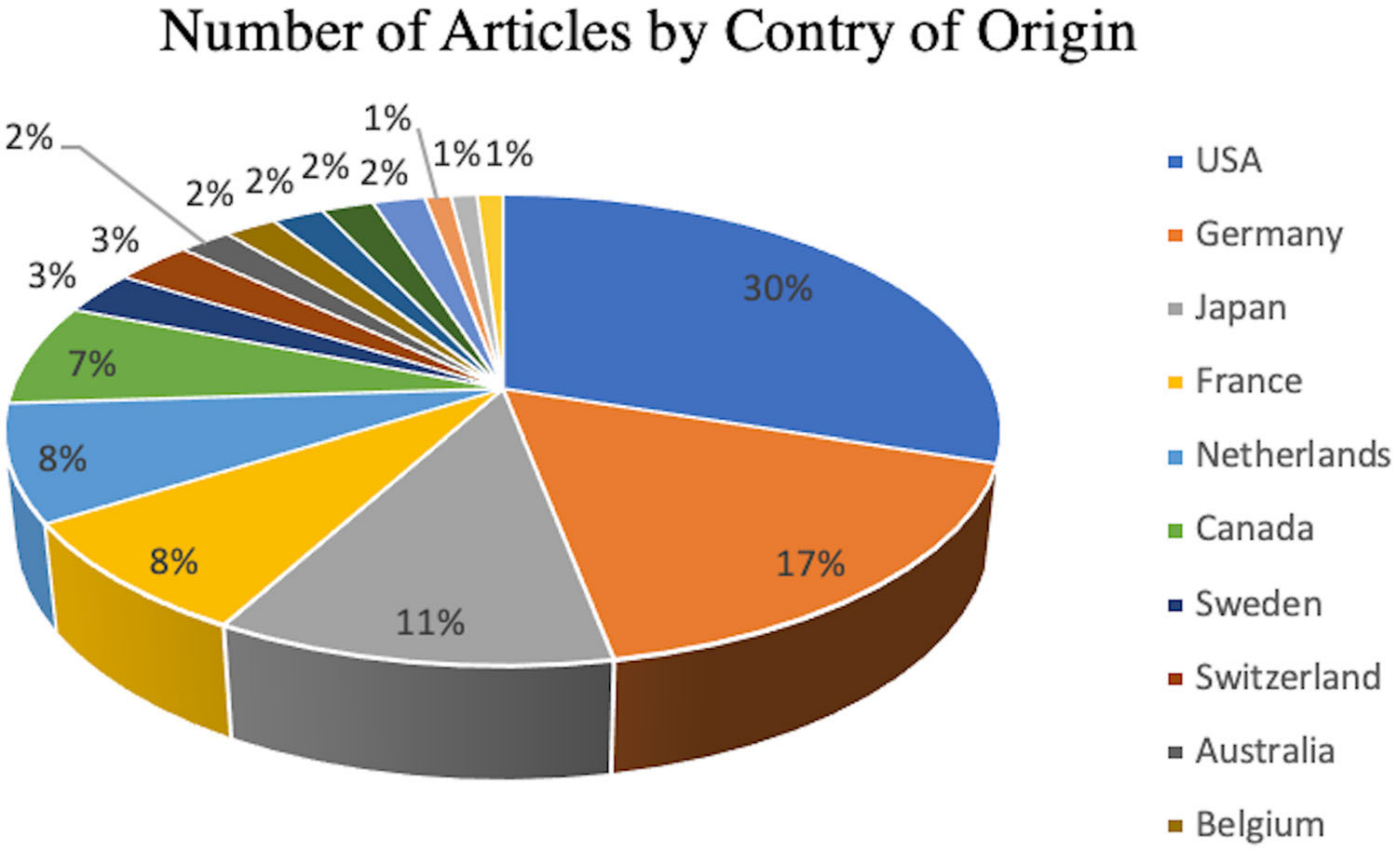
The percentage of the top 100 osteotomy around the knee publications originating from each country.

The articles were also analyzed for their author, journal, and country of origin. The top four most productive and cited authors were Lobenhoffer, P from the Unfallchirurgische Klinik (Hannover, Germany) (eight articles), Agneskirchner, JD from the Henriettenstiftung Hannover (Hannover, Germany) (six articles), Noyes, FR from the Cincinnati SportsMedicine & Orthopaedic Center (Cincinnati, USA) (six articles) and Imhoff, AB from the Hôpital Rechts der Isar (Munich, Germany) (five articles). Overall, 22 different journals were represented. The journal with the most studies from the top 100 articles was the *American Journal of Sports Medicine*, with 17 citations. Furthermore, 66 of the 100 articles were published in the top five journals (Table 2). In total, these articles represented 16 different countries of origin. The United States represented 30 of the 100 articles. Germany was second with 17 articles and Japan third with 11 articles (Figure 2).

**Table 2:** The top five journals with highest number of citations.

Rank	Journal title	Number of articles
1	*American Journal of Sports Medicine*	17
2	*Journal of Bone and Joint Surgery‐American Volume*	15
3	*Clinical Orthopaedics and Related Research*	13
4	*Arthroscopy Journal*	11
5	*Journal of Bone and Joint Surgery‐British Volume*	10
**Total**	**66**	

**Figure 3:**
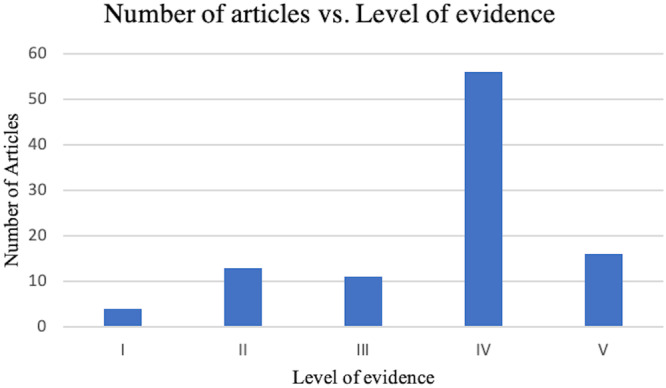
Categorization of the top 100 most‐cited knee osteotomy publications by level of evidence.

There were six different study types represented in the 100 articles: randomized control, cohort study, case series, review article, descriptive articles and laboratory study. Of these, case series design was the most used, with 55 articles. Nonrandomized controlled trials, case–control studies and case reports were not represented (Table 3). In addition, the levels of evidence used in the articles were reported (Figure 3). Level IV was the most common level of evidence, with 56 articles. The next most common was Level V, with 16 articles, followed by Level II studies (*n* = 13), Level III studies (*n* = 11) and Level I studies (*n* = 4).

**Table 3:** The study design used in the top 100 knee osteotomy publications.

Type	Number of articles
Randomized controlled trial	8
Nonrandomized controlled trial	0
Cohort study	19
Case–control study	0
Case series	55
Case report	0
Review article	4
Descriptive article	4
Laboratory study	10

## DISCUSSION

The 100 most‐cited publications in osteotomy around the knee represent a publication range from 1976 to 2015 and were cumulatively cited more than 16,000 times, with the most‐cited publication receiving 752 citations. In addition, most of the publications included in this analysis originated from the United States (*n* = 30), were case series (*n* = 55) and had Level IV evidence (*n* = 56).

Osteotomy around the knee has been reported to achieve promising results in patients with knee deformity associated with unicompartmental knee arthritis [3, 4, 5, 12, 27]. The factors that influence the incidence of knee osteotomy in an institution depend on whether the recommended indications and surgical techniques are correctly used. This bibliometric study highlights the development of osteotomies over the last 20 years and recalls the main advances that have been made, enabling us to define the following lines of research.

Older publications do not always have an advantage in total citations. The earliest publication from 1976 [24] is ranked 74th in total citations and 100th in citation density. Approximately half of the 100 most‐cited knee osteotomy publications were published in the 2000s. This probably reflects on the newer developments in knee osteotomy, such as the use of new materials (including locking plates), bone substitutes and newer technology such as PSI. This is in contradiction with other bibliometric analyses which have demonstrated a majority of cited orthopaedic publications between the 1970s and 1990s [8, 23, 26]. These corresponded essentially to the expansion of arthroscopy procedures and arthroplasty.

Looking at Table 1 one must realize that the selection of the right keywords is of major importance while searching for manuscripts. Titles of articles may not contain osteotomy as a word and still be relevant to the subject. From the table, articles listed on nos. 1, 10, 11, 22, 23, 25, 26, 30, 44, 58, 59, 61 and 63 all do not contain the word osteotomy, which implies that the osteotomy may be associated with another procedure whose clinical and scientific impact has been estimated by the authors to be greater.

The most common level of evidence was Level IV (*n* = 56), which corresponds to the most common study designs represented: Case Series (*n* = 55). Previous similar distribution of levels of evidence and type of study were reported in other orthopaedic publications [1, 21, 30]. This suggests that large impacts have been made by studies with small effects on patients, indicating their originality during the period of publication.

A limitation of this study is the correlation between a publication's impact and its total number of citations. Although the latter measure is generally accepted as most representative and more objective than others, still influential articles can be overlooked. Some subjectivity was unavoidable in making final inclusion or exclusion decisions. Despite this limitation, we reduced this subjectivity through the consensus of multiple authors. Though the Web of Knowledge database does perform quality control, we recognize that bias may be introduced at the data level by authors who cite their own works or preferentially cite from certain journals. However, citations resulting from these actions probably serve as a small fraction of the total citations, considering these 100 articles were cited more than the multitude of other articles on knee osteotomy. With the limitations discussed, citation analysis remains a widely accepted tool to measure a publication's impact on the field.

## CONCLUSION

The 100 most influential publications in knee osteotomy were cited a total of 16,246 times. The study designs most used were case series and cohort studies with low‐level evidence. This publication serves as a reference to direct orthopaedic practitioners to the 100 most influential studies in knee osteotomy and target future research directions.

## AUTHOR CONTRIBUTIONS

Grégoire Micicoi designed the protocol and performed the statistical analysis. Grégoire Micicoi, Axel Machado and Raghbir Khakha collected the data. Matthieu Ollivier and Grégoire Micicoi wrote the initial draft and performed revisions of subsequent drafts. Grégoire Micicoi, Raghbir Khakha, Jean‐François Gonzalez, Matthieu Ollivier and Ronald van Heerwaarden corrected the final draft of the manuscript. All the authors approved the submitted version.

## FUNDING INFORMATION

No funding was needed for this study.

## CONFLICT OF INTEREST STATEMENT

Matthieu Ollivier is educational consultant for Newclip, Stryker and Arthrex. Raghbir Khakha, Sachin Tapasvi and Ronald van Heerwaarden are consultants for Newclip. Jean‐François Gonzalez is consultant for Amplitude. The other authors declare no conflicts of interest.

## ETHICS STATEMENT

Due to the public nature of the exploited data, an institutional review board was not necessary for conduction of this study.

## Data Availability

Data are available on request.
